# Supplementary material for: Causal Relationship between Aging and Anorexia Nervosa: A White-Matter-Microstructure-Mediated Mendelian Randomization Analysis
Source: Biomedicines. 2024 Aug 16;12(8):1874. doi: 10.3390/biomedicines12081874 (PMC11351342; doi:10.3390/biomedicines12081874)
Supplement: Supplementary file 1 [file biomedicines-12-01874-s001.zip › Supplementary Figure/Figure S4/Singe SNP plot of MR analysis for TL to ubm-b-1917.pdf]

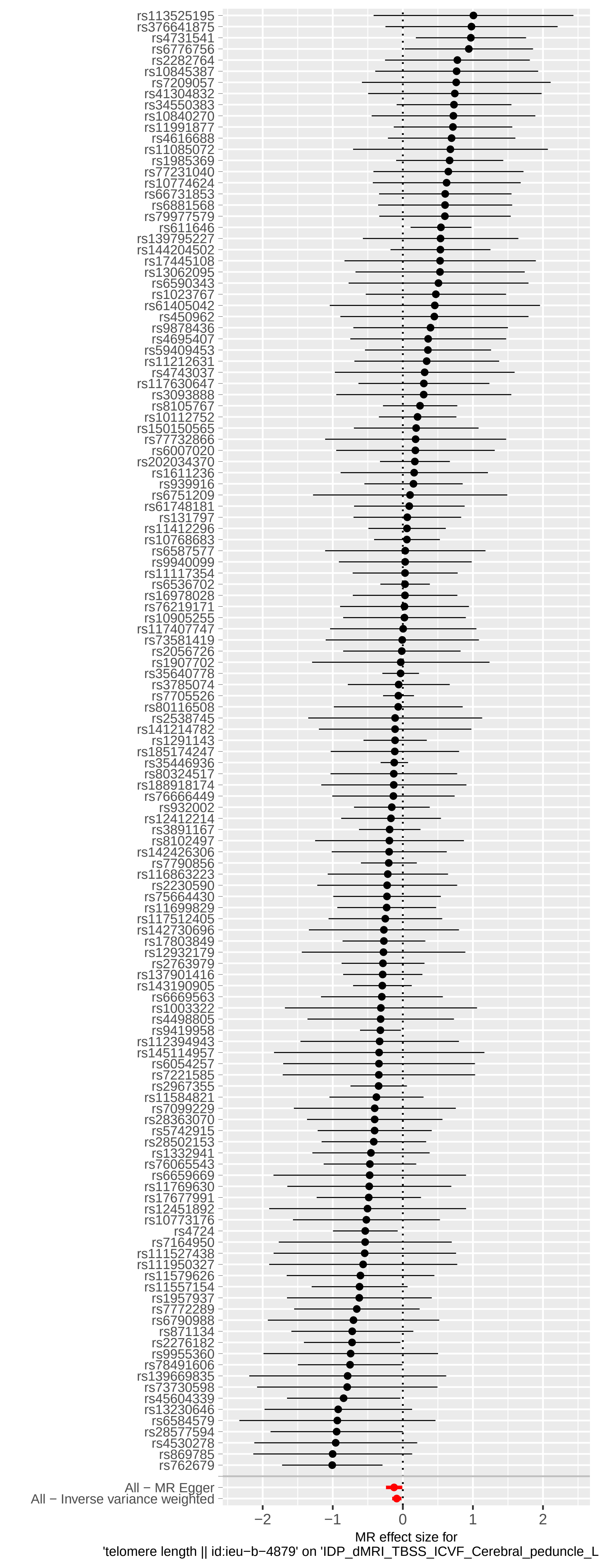
rs113525195
rs376641875
rs4731541
rs6776756
rs2282764
rs10845387
rs7209057
rs41304832
rs34550383
rs10840270
rs11991877
rs4616688
rs11085072
rs1985369
rs77231040
rs10774624
rs66731853
rs6881568
rs79977579
rs611646
rs139795227
rs144204502
rs17445108
rs13062095
rs6590343
rs1023767
rs61405042
rs450962
rs9878436
rs4695407
rs59409453
rs11212631
rs4743037
rs117630647
rs3093888
rs8105767
rs10112752
rs150150565
rs77732866
rs6007020
rs202034370
rs1611236
rs939916
rs6751209
rs61748181
rs131797
rs11412296
rs10768683
rs6587577
rs9940099
rs11117354
rs6536702
rs16978028
rs76219171
rs10905255
rs117407747
rs73581419
rs2056726
rs1907702
rs35640778
rs3785074
rs7705526
rs80116508
rs2538745
rs141214782
rs1291143
rs185174247
rs35446936
rs80324517
rs188918174
rs76666449
rs932002
rs12412214
rs3891167
rs8102497
rs142426306
rs7790856
rs116863223
rs2230590
rs75664430
rs11699829
rs117512405
rs142730696
rs17803849
rs12932179
rs2763979
rs137901416
rs143190905
rs6669563
rs1003322
rs4498805
rs9419958
rs112394943
rs145114957
rs6054257
rs7221585
rs2967355
rs11584821
rs7099229
rs28363070
rs5742915
rs28502153
rs1332941
rs76065543
rs6659669
rs11769630
rs17677991
rs12451892
rs10773176
rs4724
rs7164950
rs111527438
rs111950327
rs11579626
rs11557154
rs1957937
rs7772289
rs6790988
rs871134
rs2276182
rs9955360
rs78491606
rs139669835
rs73730598
rs45604339
rs13230646
rs6584579
rs28577594
rs4530278
rs869785
rs762679
All − MR Egger
All − Inverse variance weighted
−2
−1
0
1
2
MR effect size for
'telomere length || id:ieu−b−4879' on 'IDP_dMRI_TBSS_ICVF_Cerebral_peduncle_L
